# Non-Coding RNAs as Potential Biomarkers for Colorectal Polyps and Cancer Detection

**DOI:** 10.3390/ijms26094106

**Published:** 2025-04-25

**Authors:** Samo Plut, Aleksandar Gavric, Damjan Glavač

**Affiliations:** 1 Department of Gastroenterology, University Medical Centre Ljubljana, 1000 Ljubljana, Slovenia; samo.plut@kclj.si (S.P.); aleksandar.gavric@kclj.si (A.G.); 2 Ljubljana Digestive Endoscopy Research Group (LuDERG), Department of Gastroenterology, UMC Ljubljana, 1000 Ljubljana, Slovenia; 3 Faculty of Medicine, University of Ljubljana, 1000 Ljubljana, Slovenia; 4 Department of Molecular Genetics, Institute of Pathology, Faculty of Medicine, University of Ljubljana, 1000 Ljubljana, Slovenia; 5 Center for Human Genetics and Pharmacogenomics, Faculty of Medicine, University of Maribor, 2000 Maribor, Slovenia

**Keywords:** colorectal polyp, colorectal cancer, CRC, non-coding RNAs, epigenetic regulators

## Abstract

Colorectal cancer (CRC) remains one of the leading causes of cancer-related death worldwide. The precursor of CRC is a colorectal polyp, of which adenoma is the most common histological type. The initial step in CRC development is the gradual accumulation of a series of genetic and epigenetic alterations in the normal colonic epithelium. Genetic alterations play a major role in a subset of CRCs, but the pathophysiological contribution of epigenetic aberrations has recently attracted attention. Epigenetic marks occur early in cancer pathogenesis and are therefore important molecular hallmarks of cancer. This makes some epigenetic alterations clinically relevant for early detection not only of CRC but also of precancerous polyps. In this review we focus on three types of non-coding RNAs as epigenetic regulators: miRNA, lncRNA, and lncRNAs, highlighting their biomarker potential.

## 1. Introduction

Despite significant advances in detection and treatment through endoscopy, surgery, chemotherapy, radiotherapy, and immunotherapy, CRC remains the second leading cause of cancer-related death worldwide and is the third most common malignancy worldwide [1]. Colorectal polyps are the precursors of CRC and are present long before invasive cancer, providing opportunities to prevent cancer by removing precursor lesions. Endoscopic resection is now a core activity that reduces the incidence and mortality of CRC [2]. The development of CRC is generally accepted as a sequential process, in which genetic mutations determine the phenotypic progression of the tumour. CRC develops through the gradual stepwise accumulation of mutations leading to transformation from polyp to cancer through the adenoma–carcinoma sequence [3].

The development from adenoma to carcinoma takes an average of 5 to 15 years and is not influenced by a single pathway. It is a complex, multifactorial process characterised by chromosomal instability (CIN), microsatellite instability (MSI), and DNA methylation in CpG island regions (CIMP). All of these pathways can overlap and are responsible for genetic instability in adenomas that can lead to malignant transformation [4].

Epigenetics are heritable changes in gene expression that do not result in permanent changes in the DNA sequence and play an important role in the pathogenesis of CRC [5]. The prevailing consensus is that epigenetic alterations in cancer occur early and are more common than genetic alterations. Non-coding RNA (ncRNA) is an RNA molecule that does not code for proteins but plays a critical role in regulating gene expression. ncRNAs can modify gene transcription by interacting with DNA, RNA, and proteins. ncRNAs play a critical role in regulating the initiation and progression of several cancers, accounting for nearly 60% of the transcriptional output in human cells [6,7].

In this review, we provide an overview of the role of non-coding RNAs (ncRNAs) as one of the fundamental epigenetic modifications in CRC. We will highlight major ncRNAs (miRNAs and long ncRNAs) as epigenetic regulators. The topic of ncRNAs has grown exponentially in recent years. It is therefore impossible to cover the majority of published papers. In this article, we have focused on only a few known ncRNAs in the context of colorectal polyps and CRC. Of note, DNA methylation and histone modifications are at the core of the epigenetic modifications in CRC, but we will only briefly mention them.

## 2. Epigenetic Alterations in Colorectal Polyps and Cancer

According to the type of genomic or epigenomic instability, CRC can be divided into three distinct groups: CIN, MSI, and CIMP [7]. Another classification is based on altered gene expression, which has been compiled by the CRC Subtyping Consortium and is called the consensus molecular subtypes (CMS) of CRC [8]: CMS1 (approximately 14% of all CRCs) is associated with MSI, *BRAF* mutation, promoter hypermethylation, and immune infiltration. CMS2 (the most common subtype with 37% of all CRC cases) is associated with CIN and involves activation of the Wnt and Myc pathways. CMS3 (13% of CRCs) is characterised by metabolic dysregulation and KRAS mutation. CMS4 (23% of CRCs) is described as a mesenchymal, stroma-rich group, with prominent transforming growth factor beta activation, stromal invasion, and angiogenesis. Downregulation of the miR-200 family is associated with CMS4 tumours [6].

Irrespective of the CRC development pathway, epigenetic alterations are present in virtually all CRCs [9,10]. Of the epigenetic modifications found in colorectal polyps and CRC, aberrant DNA methylation is the best studied and appears to play a role in both polyp formation and progression to CRC. CpG island DNA methylation affects CpG-rich regions (called CpG islands) in the 5′ region of genes and results in transcriptional silencing of tumour suppressor genes [11]. Initially not identified as precancerous, SSLs have CIMP and CIMP CRC arises almost exclusively from SSLs [7]. Alterations in chromatin structure or histone modification states are inconclusive—increased expression of HDAC2 and increased acetylation of H4K12 and H3K18 were found in adenomas compared to normal colon mucosa, but little else is known [7].

Regarding ncRNAs, miR-143 and miR-145, which are usually co-expressed, are often downregulated in the early phase of adenoma formation and not during CRC progression. Hence, these two miRNAs do not seem to have clinical prognostic potential but could be promising biomarkers for early diagnosis [12].

Members of the miR-34 family have been described as tumour suppressors, and miR-34a appears to play an important role in the loop with p53 [13]. Both miR-34b and miR-34c are associated with metastasis and poor prognosis, suggesting that they play a role not only in the development of cancer but also in its progression [14]. miR-143 and the miR-34 family are considered as tumour suppressors [6]. Another example of a well-studied miRNA is miR-21 [12], which plays a role in several pathways, in particular the AKT pathway [15] and *PDCD4* gene silencing. Another is miR-31, which may contribute to the activation of the RAS signalling pathway [16]. As the RAS pathway is the main effector pathway for EGF, miR-31 has been shown to reduce response to EGFR antagonist treatment in CRC [12]. Both miR-21 and miR-31 are oncogenic miRNAs.

## 3. Non-Coding RNAs

In less than three decades since their discovery, several thousand ncRNAs have been identified and can be categorised according to their size or function [17]. One of the best-known types of ncRNA is microRNA (miRNA). Another type of ncRNA is long non-coding RNA (lncRNA), which is longer than miRNAs and regulates gene expression by interacting with DNA-binding proteins. Other subtypes of ncRNA include circular RNAs (circRNAs), small nuclear RNAs (snRNAs), and small nucleolar RNAs (snoRNAs). These are not described in detail in this report.

Many ncRNAs are shed from cancer cells into the blood or urine and act as diagnostic markers or prognostic indicators; ncRNA in tumour tissue can provide additional information and act as a predictive biomarker. Biomarkers are molecular patterns that can be used as a key tool for early detection, prognostication (assessment of metastasis risk and adjustment of both surveillance and treatment), and prediction of treatment response (tailored treatments according to tumour molecular subtype) [18].

### 3.1. miRNA

Since their discovery in 1993, this class of ncRNAs has been the most extensively studied and best characterised [19]. Due to their relative stability compared to endogenous RNAs, miRNA expression can be detected in plasma, stool, urine, saliva and tissue samples [18].

miRNAs regulate the translation of over 60% of protein-coding genes, including those involved in key pro-tumorigenic processes such as cell proliferation, differentiation, and apoptosis. They function either by regulating specific individual target mRNAs or by acting as broad regulators of gene expression with a high degree of functional redundancy (multiple miRNAs have been shown to regulate a single target mRNA as well as mediating the expression of hundreds of genes simultaneously) [6,20].

miRNAs are involved in regulating the pathogenesis of cancer and pre-cancerous lesions by exerting oncogenic and tumour suppressor functions through regulation of translation, affecting cell growth, differentiation, and apoptosis. Elevated serum plasma levels of miR-92a and miR-29a are associated with advanced adenomas with 62–64% sensitivity and 84–81% specificity [21]. Expression of miR-21, miR-29a, and miR-125b is associated with early CRC, as well as tubular adenomas and high-grade intraepithelial neoplasia [22]. Several studies have shown that miRNAs are a useful prognostic marker (associated with higher TNM stage, lymph node, and peritoneal metastasis) and a good predictor of treatment response (associated with poor chemotherapy response and chemoresistance to various chemotherapy and immunotherapy regimens) [23].

Selected miRNAs and also lncRNAs play an important role in all major signalling pathways relevant to CRC [6].

Activation of the RAS-RAF-MEK pathway, which can modulate treatment response, may occur via downregulation of miR-143 [12] and/or upregulation of miR-31 [16]. Another extensively studied miRNA in CRC is miR-21, which may promote cancer progression through hyperactivation of the phosphoinositide 3-kinase (PI3K)-AKT pathway [15]. It has been shown that miR-21 can target programmed cell death protein 4 (PDCD4), which is thought to be an important mediator in apoptosis effector pathways [12]. Expression of miR-34a enhances p53 activity, thus increasing tumour suppressor activity. In addition, miR-34a is involved in transforming growth factor-β (TGF- β) signalling, resulting in increased cell invasion [16]. In addition, miR-29a has been reported to decrease the expression of E-cadherin [15] in epithelial cells, the loss of which favours cell growth, migration, and invasion. miR-135 downregulates APC, which plays a role in activation of WNT-β-catenin pathway [24]. New blood vessel formation is critical for tumour survival and is regulated by the vascular endothelial growth factor (VEGF) signalling pathway. VEGF is a direct target of miR-126, which is frequently downregulated in CRC [25]. One study showed that downregulation of miR-126 was associated with worse outcomes in patients with metastatic CRC treated with the anti-VEGF antibody bevacizumab [26].

### 3.2. lncRNA

lncRNAs are located within the intergenic region of the genome and are heterogeneous, not highly evolutionarily conserved (only 5–6% of lncRNAs have conserved sequences). They are longer than 200 nucleotides. Depending on their function, they act as positive or negative regulators of transcription by interacting with gene promoters or enhancers, modifying chromatin access, regulating nuclear architecture, directly interacting with target mRNAs and regulatory protein complexes (which regulates mRNA stability), and agglutinating miRNAs through multiple specific binding sites [6]. Depending on their proximity to the protein-coding region, lncRNAs are classified as sense and antisense lncRNAs, intronic lncRNAs, bidirectional lncRNAs, and long intergenic ncRNAs [23]. lncRNAs can be also classified by their location; cytoplasmic, nuclear, and cytoplasmic nuclear [27]. Another classification is by their molecular function: guide, decoy, and backbone molecules [28]. Some lncRNAs have oncogenic features, while others have tumour suppressor roles [29]. In combination with mi-R29a, the lncRNA *HOX* leads to a loss of function of E-cadherin [30]. LncRNA *GAS5*, which together with miR-126 targets VEGF, is often downregulated in CRC [31].

## 4. Biomarker Role of Epigenetic Alterations

Colonoscopy is the gold standard for CRC screening because it can detect, and often remove, precancerous polyps. The disadvantages of colonoscopy are its high cost, the need for trained endoscopists, and the invasiveness of the procedure for the patient. This is a major reason for the somewhat low compliance rate. Finally, although rare, a diagnostic colonoscopy can be associated with complications such as bleeding and perforation [15]. The main limitation of the non-invasive faecal immunochemical test is its lower sensitivity and specificity for precursor lesions such as adenomas, compared with colonoscopy [15]. As a result, there is a substantial proportion of unnecessary colonoscopies [32]. The need for less invasive strategies to detect both precancerous lesions and early-stage CRC is an important clinical priority, as is the need for more efficient ways of triaging patients for colonoscopy. Currently, numerous ncRNA candidates are being investigated as diagnostic biomarkers for CRC, with the majority being blood- and stool-based. Some tissue-based ncRNAs may have better prognostic biomarker potential rather than diagnostic [6]. Some ncRNA candidates as diagnostic biomarkers in CRC are as follows: miR-21, miR-92a, and miR-29a are blood-based and could be used as diagnostic biomarkers for adenoma detection, while miR-21 and miR-92a are stool-based. Potential blood-based ncRNAs for CRC detection are miR-21, miR-92a, miR-29a, miR-20a, and miR223 in the miRNA group, while HOTAIR, CCAT1, and CRNDE are potential lncRNAs. The following miRNAs have potential as stool-based biomarkers for CRC detection: miR-21, miR-92a, miR-20a, and miR-223 [6]. Of note, before ncRNAs can be used in clinical practice, large-scale validation studies are required.

### 4.1. miRNAs as Potential Biomarkers for Polyp and CRC Detection

miRNAs are stable in tissue, blood, and stool; they are small in size and limited in their number. These properties make them good candidates as diagnostic biomarkers [33]. Regarding the biomarker role for diagnosis, the analysis of miRNAs in stool samples has attracted lots of interest. CRCs secrete miRNAs (and other ncRNAs) directly and continuously into the intestinal lumen [16]. The levels of miRNAs are also altered in certain pathological conditions [34], in the presence of precursor lesions [35], and during the development of CRC [36]. This makes them even more attractive targets for biomarker research. Surprisingly, one study found miR-320a, let-7b-5p, and let-7a-3p more abundant in the stool of CRC patients; these same miRNAs were more expressed in adjacent mucosa than in tumour tissue [16]. Although the number of published papers investigating miRNAs in CRC has increased significantly, there is a lack of well-designed studies. In addition, none of the biomarkers have met the requirements for clinical translation, namely a cohort of >1000 individuals, prospective design, and comparison with established screening and diagnostic methods. Jung et al. conducted a comprehensive literature search and collectively analysed 15,839 CRC specimens and 453 colorectal adenomas. They identified the following miRNAs that are relevant for biomarker research: miR-21, miR-143, miR-145, miR-194, and the miR-200 family [6]. Studies have shown that upregulated miR-21 expression is a promising biomarker not only for diagnosis but also for prognosis and prediction of treatment response in CRC. miR-21 has demonstrated high sensitivity and specificity in blood and stool, although results from the stool analysis showed a higher level of variability [6]. miR-21 expression in blood showed a sensitivity and specificity of up to 80% for the diagnosis of colorectal adenomas [37]. miR-92a is also studied as an adenoma biomarker. The study showed that sensitivity was higher when using blood rather than stool samples [38]. To increase diagnostic accuracy, panels of several ncRNAs are being used simultaneously. A four-miRNA panel (miR-19a-3p, miR-223-3p, miR-92a-3p, and miR-422a) in serum samples was able to distinguish patients with adenoma from those with CRC with AUC of 0.87 [39]. In a recently published study [16], the authors showed altered levels of twenty-five miRNAs in the stool of CRC patients. Notably, a 5-miRNA signature including miR-149-3p, miR-607-5p, miR-1246, miR-4488, and miR-6777-5p was able to distinguish patients from control individuals. Interestingly, tissue miRNA profiles mirrored those of stool samples.

A recent study showed increasing expression (tissue-based) of miR-378i from adenoma to CRC [40]. Hypothetically, such miRNAs could serve as biomarkers of cancer invasion and could be a novel marker of tumour invasiveness that could be useful for treatment planning (endoscopic vs. surgical resection of early cancers). Some of the promising miRNA biomarkers for non-invasive diagnosis of CRC and adenoma are summarised in Table 1. The most promising miRNA biomarker panels for non-invasive diagnosis of adenomas in blood are summarised in Table 2.

### 4.2. lncRNAs as Potential Biomarkers for Polyp and CRC Detection

Less is known about lncRNAs than about miRNAs, and the discovery of lncRNAs is still ongoing. Their function is not yet fully understood so the number of lncRNAs with relevant functions in cellular physiology remains unclear. To date, fewer than 100 different lncRNAs associated with CRC have been listed in the NONCODE database [55] and, unlike miRNAs, more lncRNAs are tissue-derived compared to blood-derived lncRNAs [6]. *HOTAIR* was found to be upregulated in the early stages of CRC development. It has also been associated with poor patient survival [56]. Another one is *CCAT1*. Its upregulation seems to be an early event in colorectal carcinogenesis and was associated with overall poor survival [57]. It has an oncogenic role by activating the Myc signalling pathway [58]. *GAS5* is downregulated in human CRC tissues in comparison to tumour-adjacent normal tissues [57]. Low levels of *GAS5* were positively correlated with large tumour size and poor overall survival [57]. Expression of *CASC11* correlates with the tumour size [59]. It was reported that *CASC11* suppresses the Wnt signalling pathway in CRC [60]. Another lncRNA that interferes with the Wnt signalling pathway is *CCAT2* [61]. At present, most of the research on lncRNAs is still focused on discovery and functional aspects. We have summarised some lncRNAs as biomarkers for diagnosis in tissue and blood in Table 3.

## 5. Conclusions

Along with DNA methylation and histone modification, ncRNAs are at the cornerstone of epigenetics. Despite the increased research in the field of ncRNAs in CRC, well-designed studies enabling clinical translation are still lacking. Hypothetically, the possibilities for using ncRNAs in clinical practice are numerous. They could serve as biological biomarkers with higher sensitivity for identifying individuals with precancerous colorectal polyps, as the FIT stool test has room for improvement in terms of sensitivity and specificity. Stool and blood samples could be used for such detection. This could help reduce healthcare costs and avoid unnecessary colonoscopies. In addition, tissue-based ncRNAs may serve as predictive biomarkers for faster polyp growth or as predictive markers for deep invasion in early CRC. This could help to better plan endoscopic therapy for colorectal polyps and early CRC. With a better understanding of the molecular drivers of polyp growth, we could more efficiently plan surveillance colonoscopies at different time intervals, which is another clinical implication. Finally, ncRNAs have the potential to serve as predictive biomarkers for specific oncological treatments for CRC. Hopefully, we will see more translational research, especially with miRNAs, because lncRNAs are still in the phase of basic research.

## Figures and Tables

**Table 1:** Summary of the most promising miRNA biomarkers for non-invasive detection of CRC or adenoma in blood and stool.

Micro-RNA	Up/Down Regulated	Test	Samples	% Sensitivity	% Specificity	References
miR-221	Up	CRC	Blood	86	41	[41]
miR-21	Up	CRC	Blood	82.8	90.6	[37]
miR-21	Up	Adenoma	Blood	73.1–76.8	68.1–81.1	[37,38,42]
miR-106a	Up	CRC	Blood	62.3	68.2	[43]
miR-18a	Up	CRC	Blood	/	/	[44]
miR-29a	Up	CRC	Blood	/	/	[44]
miR-29a	Up	Adenoma	Blood	62.2–72	66–84.7	[38,39]
miR-183	Up	CRC	Blood	73.7	88.5	[45]
miR-92a	Up	CRC	Blood			[42,46,47]
miR-92a	Up	Adenoma	Blood	64.9–65.4	78.7–81.4	[38,39,42]
miR-135b	Up	Adenoma	Stool	65	45	[48]
miR-135b	Up	CRC	Stool	78	68	[48]
miR-223	Up	CRC	Blood	46	/	[49,50]
miR-223	Up	CRC	Stool	60–76.5	71–96.4	[50,51]
miR-143/miR-145	Down	CRC	Blood	/	/	[52]
miR-143/miR-145	Down	CRC	Stool	/	/	[53]
miR-31	Up	CRC	Blood	/	/	[54]

**Table 2:** The most promising miRNA biomarker panels for non-invasive diagnosis of adenomas in blood.

Panels	% Sensitivity	%Specificity	References
miR-19a-3p, miR-223-3p, miR-92a-3p, and miR-442a	**/**	**/**	[47]
miR-532-3p, miR-331, miR-195, miR-17, miR-142-3p, miR-15b, miR-532, and miR652	88	64	[21]
miR-29a and miR-92a	73	80	[39]
miR-21 and miR-92a	70	70	[42]

**Table 3:** Summary of evidence for lncRNAs as biomarkers for diagnosis in tissue and blood.

lncRNA	Up/Down Regulated (Role)	Samples	Comment	References
H19	Up (oncogene)	Tissue (CRC)	Upregulated in CRC vs. normal tissue	[62]
HOTAIR	Up (oncogene)	Tissue (CRC)	Upregulated in CRC vs. normal tissue	[50]
HOTAIR	Up (oncogene)	Blood	Upregulated at an early stage CRC	[50]
CCAT1	Up (oncogene)	Tissue (CRC)	Upregulated in CRC vs. normal tissue	[57]
CCAT1	Up (oncogene)	Blood	Upregulated in CRC vs. healthy controls	[50]
CRNDE	Up (oncogene)	Tissue (CRC)	Upregulated in CRC vs. normal tissue	[63]
CRNDE	Up (oncogene)	Blood	Upregulated in serum exosomes of CRC vs. healthy controls	[64]
MALAT1	Up (oncogene)	Tissue (CRC)	Upregulated in CRC vs. healthy controls	[65]
GAS5	Down (tumour suppressor)	Tissue (CRC)	Downregulated in CRC vs. normal tissue	[66]
CCAT2	Up (oncogene)	Tissue (CRC)	Upregulated in CRC vs. normal tissue	[57]
BANCR	Downregulated?	Tissue (CRC)	Downregulated in CRC vs. normal tissue	[67]
PVT1	Up (oncogene)	Tissue (CRC)	Upregulated in CRC vs. normal tissue	[68]
XIST	Up (oncogene)	Tissue (CRC)	Upregulated in CRC vs. normal tissue	[69]
UCA1	Up (oncogene)	Tissue (CRC)	/	[70]
MEG3	Down (tumour suppressor)	Tissue (CRC)	/	[71]
ncRAN	Down (tumour suppressor)	Tissue (CRC)	/	[72]
ncRuPaR	Down (tumour suppressor)	Tissue (CRC)	/	[73]

## Data Availability

No new data were created or analysed in this study.

## Abbreviations

DNA	deoxyribonucleic acid
RNA	ribonucleic acid
ncRNA	non-coding RNA
miRNA	microRNA
lncRNA	long non-coding RNA
mRNA	messenger RNA
circRNA	circular RNA
snRNA	small nuclear RNA
snoRNA	small nucleolar RNA
CRC	colorectal cancer
APC	adenomatous polyposis coli
CIN	chromosomal instability pathway
CIMP	CpG island methylator phenotype
MSI	microsatellite instability
PDCD4	programmed cell death protein 4
TGF- β	transforming growth factor-β
PI3K	phosphoinositide 3-kinase
VEGF	vascular endothelial growth factor
CMS	consensus molecular subtypes
EGFR	epidermal growth factor receptor
